# Estimation of the level of anthropogenic impact based on the determination of radionuclides and heavy metals in sediments taken from Rybnik reservoir, Poland

**DOI:** 10.1007/s11356-024-33709-9

**Published:** 2024-05-21

**Authors:** Katarzyna Szarłowicz, Agnieszka Baran, Karolina Wójs, Sylwia Wójcik

**Affiliations:** 1 Faculty of Energy and Fuels, AGH University of Krakow, Krakow, Poland; 2 Department of Agriculture and Environmental Chemistry, University of Agriculture in Krakow, Krakow, Poland

**Keywords:** Radionuclides, Gamma spectrometry, Heavy metals, Sediments, Geochemical ecotoxicological indicators

## Abstract

The aim of the study was to estimate the degree of anthropogenic risk by evaluating the level of the contamination of sediments collected from the Rybnik reservoir. The results of the determination of radionuclides (^137^Cs, ^40^K, ^228^Th, ^228^Ra, ^226^Ra, ^210^Pb, ^238^U) and heavy metals (Zn, Cd, Pb, Cu, Cr, Ni) were presented. The Rybnik reservoir is located in a highly urbanised area, the Lower Silesian Voivodeship in Poland. Radionuclides (^137^Cs, ^40^K, ^228^Th, ^228^Ra, ^226^Ra, ^210^Pb, ^238^U) were measured using gamma spectrometry. The heavy metal (Zn, Cd, Pb, Cu, Ni and Cr) content was determined using an inductively coupled plasma optical emission spectrophotometer (ICP-OES). The classification of sediment pollution was made on the basis of geochemical and ecotoxicological indices. Radioactivity was varied with the highest for ^40^K (more than 200 Bq·kg^−1^). The concentrations for the remaining radionuclides were mostly below 20 Bq·kg^−1^. At the inlet zone (no. 9) an increase in radioactivity of each radioisotope was observed. The values of heavy metals from the lowest to the highest total amount in the sediments were as follows: Cd < Cr < Pb < Ni < Cu < Zn. The sediments of the reservoir are largely contaminated with Cu, but the sediments generally are contaminated to an average degree. Most pollutants accumulate in the inlet zone and near the dam wall. The content of artificial radionuclides, as well as the geochemical and ecological indicators used, can serve as an indicator of the level of anthropopressure in the vicinity of the Rybnik reservoir.

## Introduction

**Fig. 1:**
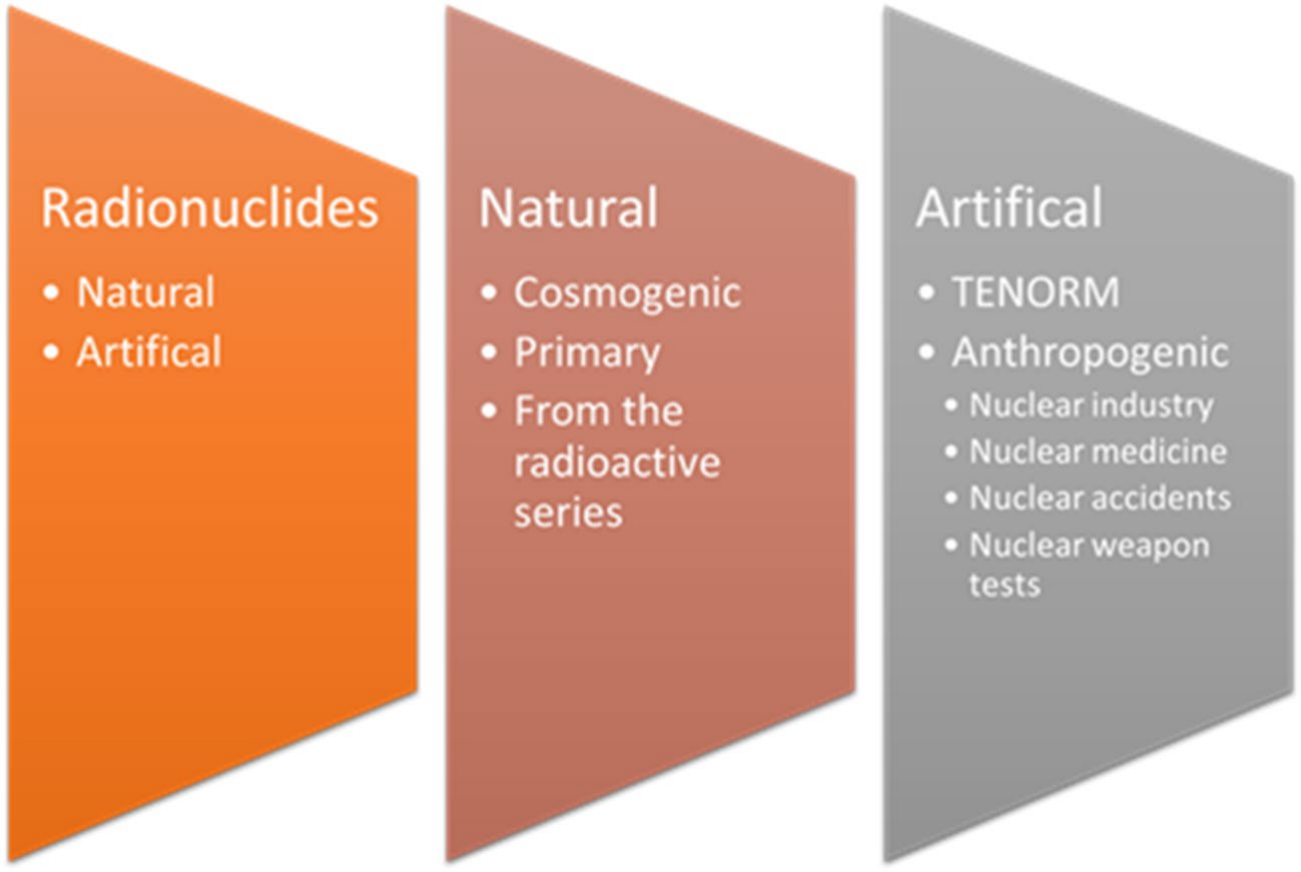
Sources of radionuclides in the environment

Pollutants, such as radionuclides, heavy metals and other types of chemical compounds can be divided into natural and artificial origins and are widely distributed in all elements of the environment. The residence time in the environment depends on the half-life and the processes that take place in the environment. In Fig. 1, the main sources of radionuclides are presented. The natural origin of radionuclides is divided into those from radioactive series, including ^235^U, ^238^U or ^210^Pb then to cosmogenic radionuclides that were created under the influence of cosmic radiation: ^14^C, ^3^H, ^22^Na and primordial ones such as ^40^K and ^87^Rb, described as single very long-lived radionuclides (Lehto and Hou 2010).

The artificial origin of radionuclides is divided into anthropogenic and TENORM (Technologically Enhanced Naturally Occurring Radioactive Material). Since 1934, when the first artificial nuclear reaction was carried out, radionuclides have been used in many fields of science, technology and medicine. And their use and disposal are carried out in accordance with radiological protection standards. However, situations where radionuclides are introduced into the environment in an uncontrolled manner pose a threat such as those originating from nuclear accidents (e.g. ^137,134^Cs, ^131^I) and nuclear weapon tests (^137^Cs, ^238^Pu). A serious accident occurred in Fukushima (March 11, 2011); the earthquake and tsunami caused a serious accident at the Fukushima Dai-ichi nuclear power plant on the northeast coast of Japan. The world’s worst nuclear disaster occurred in Chernobyl (1986), in Ukraine. Both accidents were classified at 7 (the highest) level, according to the International Nuclear and Radiological Event Scale (INES). The intensity of nuclear weapons testing occurred at the turn of the 1950s and 1960s (Fuma et al. 2017; Pravalie 2014; Voitsekhovich et al. 2007).

Many industrial sectors can be distinguished that generate TENORM, for instance, mining, energy production, water treatment, fertiliser and fertiliser production waste. As a result of technological processes, radionuclides are concentrated and redistributed into the environment. For example, the level of ^226^Ra,^230^Th or ^210^Pb so naturally occurring radionuclide is often much higher (even 20 times) in waste material from a given industry, fly ashes after coal combustion or in phosphogypsum (Baxter 1996; Nabhani et al. 2016).

Heavy metals such as Cu, Cd, Ni, Pb and Zn are often tested for their content in the environment because they have a significant impact on the functioning of the ecosystem. Heavy metals are naturally occurring elements; they are found throughout the earth crust at determined levels. Their toxicity depends on several factors, including the dose, route of exposure and chemical species, as well as the age, gender, genetics and nutritional status of the exposed individuals. Because of their high degree of toxicity, Cd, Cr, Pb and Hg rank among the prioritising metals of public health importance. Aquatic ecosystems are particularly taken into account (Kadim and Risjani 2022; Rosinska and Dąbrowska 2011).

The cycle of radionuclides and heavy metals that move through the environment is complex. Radionuclides and metals enter biogeochemical cycles and move through individual elements of the environment. They fall to the earth’s surface and through surface and underground runoff to various water reservoirs. The intensity of migration is influenced by many factors, e.g. geochemical, physical–chemical and climatic. Natural water reservoirs have greater possibilities of self-purification after the dispersion of metallic elements, but this condition is not always met. Self-cleaning is difficult or completely absent in artificial water reservoirs where pollutants disperse and accumulate. Toxic substances settle at the bottom of the tank and accumulate in the bottom sediments. The dispersion and accumulation of heavy metals and radionuclides in high concentrations pose a threat to the ecosystem, and hence, the amount of them should be systematically monitored to prevent the creation of toxic conditions for living organisms and the entire ecosystem or their further spread (Perliceusz et al. 2015; Sojka and Jaskuła 2022; Rodriguez et al. 2023, Aleksander-Kwaterczak et al. 2021).

Bottom sediments play a fundamental role in the accumulation of pollutants in water reservoirs. On the one hand, bottom sediments collect radionuclides and heavy metals, but on the other hand, they remain part of the water body. Thanks to the very good sorption properties, bottom sediments can be used to estimate the value of heavy metals and radionuclides in water reservoirs (Szarłowicz et al. 2011, 2018). The origin of anthropogenic lakes is related to the direct or indirect effects of human activity. These include dam reservoirs, dyke reservoirs, post-mining reservoirs and various types of pools in industrialised zones. These types of reservoirs have a number of functions, including retention, flood protection and recreation. Artificial water reservoirs are also often built in coal power plants to serve as a cooling element. However, there is an interaction between the reservoir and the power plant. The water in the reservoir is more exposed to contamination because, in addition to environmental pollutants, there is also the supply of additional elements from a working power plant, especially in the form of post-cooling wastewater. These elements can be harmful in large quantities or even pose a threat to the life of organisms inside and outside the tank. Pollutants are by-products produced in the energy production process. The main threat comes from the combustion of coal or biomass. By-products include fly ash, boiler slag and flue gas desulfurization residues (Uliasz-Bocheńczyk et al. 2015; Gera et al. 1991; Astatkie et al. 2021).

The aim of the study was to estimate the degree of anthropogenic risk by assessing the level of contamination of sediments collected from the Rybnik reservoir. The results of the determination of radionuclides (^137^Cs, ^40^K, ^228^Th, ^228^Ra, ^226^Ra, ^210^Pb, ^238^U) and heavy metals (Zn, Cd, Pb, Cu, Cr, Ni) were presented. These data were used to interpret the indicators that formed the basis of anthropopressure. The way of interpreting the data and the achieved effects constitute a reference point for discussion in a global aspect. The Rybnik reservoir is located in the south-west part of the Lower Silesian Voivodeship in Poland. The reservoir is a particularly vulnerable place due to its location and operation. The reservoir’s water resources are mainly used to cool the units and turbines of the nearby power plant. The water has little or even no possibility of flow; hence, the water in the tank is constant. Potential pollutants from the power plant enter directly into the water. Furthermore, it is a highly urbanised place with many industrial branches: coal mining, metallurgy, energy and production of machines and chemicals (Kostecki and Kowalski 2004). Therefore, it is an excellent object to confirm the research thesis that bottom sediments are an ideal indicator of the assessment and extent of anthropopressure of a given ecosystem.

## Material and methods

### Study area and sampling

**Fig. 2:**
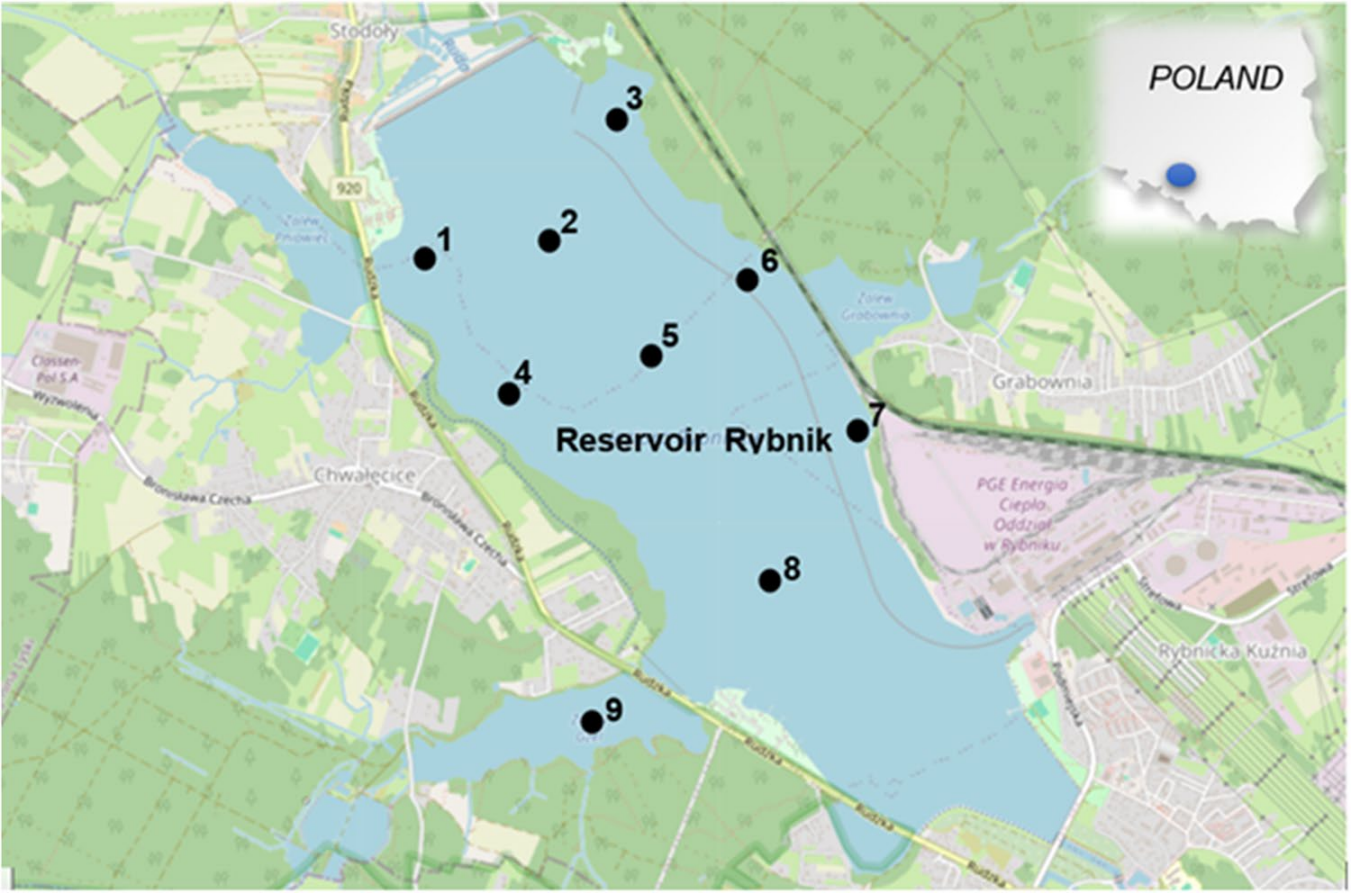
Rybnik Reservoir—location of bottom sediment sampling (OpenStreetMap Licence – CC BY-SA 2.0)

The Rybnik reservoir in Poland is located in the area of the anthropogenic lake district, which forms clusters of anthropogenic reservoirs in Upper Silesia. The Silesian area is one of the most industrialised areas in Poland. It is an anthropogenic dam reservoir created in 1972 as a result of damming the waters of the Ruda River by an earth dam (Kostecki and Kowalski 2004). It is situated in the southwestern part of the Lower Silesian Voivodeship in the northern district of Rybnik called Stodoły (Fig. 2). The reservoir is an integral element of the Rybnik Power Plant, which is located on its eastern shore. The water resources collected in the reservoir are mainly used to cool power units and turbines. The Rybnik reservoir serves as a direct receiver of purified industrial sewage and heated cooling water that comes from the power plant after the technological process. It is fed by the polluted waters of the Ruda River and, during floods, also the Nacyna River (currently bypassing the reservoir). The Ruda River itself is also a receiver of municipal sewage coming from municipal treatment plants and other industrial plants (Baran and Tarnawski 2015). Apart from its industrial role, the Rybnik reservoir also has socio-economic importance (Kostecki and Kowalski 2004). It is a place for relaxation and recreational water sports. There are sports centres and clubs nearby, as well as a recreation complex. This lake is also a popular place to fish.

The samples were collected using an Eckman corer. There were nine sampling points in the Rybnik reservoir (Fig. 2). The samples were taken to a depth of 0–15 cm in 2017. In the laboratory, the sediments were air-dried, sieved and homogenised. Detailed information on the sampling methodology is provided in the article (Baran et al. 2019). Based on the particle size fraction, the sand fraction (56–99%) was dominant in the bottom sediments sampled from the Rybnik reservoir.

### Radionuclide determination

Radiation measurements were performed using a high-resolution gamma-ray spectrometry technique. The detection system consisted of a high-purity germanium detector (model type BE3830) with a cryostat located in a Dewar vessel and electronic (high voltage, pre-amplifer, amplifer, multi-channel analyser (MCA)) and computer. The relative efficiency of the detector is equal to 34%. The spectrometer was equipped with shielding consisting of lead, cadmium and copper to reduce the background. The spectrum was analysed using Genie 2000 software (made by Canberra). Sediment samples were enclosed in a special container with a volume of 27 cm^3^. The measuring vessel was filled with very careful care to fill its entire surface. Samples packed in this way were weighed; their bulk density was determined and then sealed with parafilm. In this form, the samples were left for a month to establish the radioactive equilibrium between ^226^Ra and ^222^Rn radionuclides. After this time they were placed on the detector, and each sample was measured at least 72 h. Gamma-ray spectra were analysed, and quality and quantity analyses were performed. The radioactivity (A) of each radionuclide was calculated using the equation:

$\normalsize A=\frac{\text{}\left(CPS_{s}-CPS_{b}\right)\cdot1000}{\text{}m\cdot{P_{\gamma}\cdot\epsilon\cdot{T_{\gamma}}}}$where CPS_s_ is counts per second for the sample, CPS_b_ is counts per second for the background, m is mass of the sample, *P*(ɣ) is probability of emission, (ℇ) is efficiency and *T*_ɣ_ is self-absorption coefficient.

Measurement uncertainties were determined using the complete differential method. The radioactivity concentrations of ^226^Ra, ^228^Ra, ^228^Th, ^40^K, ^210^Pb, ^238^U and ^137^Cs were measured in the sediment samples. The corresponding peak energy values were used to identify the radionuclides. The radioactivity concentration of ^226^Ra was obtained from the determinations of ^214^Pb (295.3; 351.9 keV) and ^214^Bi (609.3 keV), whereas the ^228^Th was determined by ^208^Tl (583.4 keV; 2614.5 keV) and ^212^Bi (727.3 keV) and ^212^Pb (238.6 keV). ^228^Ra was identified by ^228^Ac (911.2; 338.3 keV). For ^238^U, the energies of 63.3 keV and 92.3–92.8 keV were used. The rest of radionuclide determination was made directly using the line from them as follows 46.5 keV (^210^Pb), 1460.8 keV (^40^K) and 661.6 keV (^137^Cs).

### Heavy metal content determination

The total metal content (Zn, Cd, Pb, Cu, Ni, Cr) in the bottom sediments was assessed after hot digestion in a mixture of HNO_3_ and HClO_3_ (3:2 v/v) acids (suprapure, Merck). The element content in the obtained solutions was determined using an inductively coupled plasma optical emission spectrophotometer (ICP-OES)—PerkinElmer Optima 7300 DV (PerkinElmer, Inc., Waltham, MA, USA). The data obtained were calculated in relation to 1 kg of dry sediment mass.

### Geochemical and ecotoxicological indices

The classification of metal-contaminated sediments was based on the Bojakowska geochemical quality classes of bottom sediments (2001), as well as the calculated geoaccumulation index (*I*_geo_) and sediment quality guidelines (SQG). Geoaccumulation index (*I*_geo_) was expressed by the formula:

$$I_{geo}=log_{2}\cdot\left[\frac{C}{1.5\cdot{B}}\right]$$

where C is the metal content in sediment, 1.5 is constant, allowing for the analysis of fluctuations in metal content as a result of natural processes and B is metal content in reference background (Bojakowska and Sokołowska 1998).

The geochemical background as assessed by the Polish Geological Institute (Bojakowska and Sokołowska 1998) was used, i.e. 0.5 for Cd, 6 for Cr and Ni, 7 for Cu, 15 for Pb and 73 for Zn (values in mg·kg^−1^ dry).

The ecological assessment was carried out using the method of numerical indicators of sediment quality: TEC, PEC and MEC. TEC (Threshold Effect Concentration) indicates the threshold value used to identify the concentration of pollutants below which there is no toxic effect on benthic organisms. The PEC (Probable Effect Concentration) indicates the probable value, which gives the concentration at which, if exceeded, it may cause negative impacts on benthic organisms. MEC (Midpoint Effects Concentration) indicates the concentration of a pollutant which is the average value between the concentrations specified by the threshold values of the TEC and PEC indicators (MacDonald et al. 2000).

## Results and discussion

### Radionuclides determination

The level of radioactivity of natural (^226^Ra, ^228^Th, ^228^Ra, ^40^K,^210^Pb, ^238^U) and artificial (^137^Cs) radionuclides was analysed in selected sediment samples. Results were presented in the units of Bq·kg^−1^ as radionuclide concentration. The results are presented in Table 1. Most of the radionuclides found in the bottom sediment of the Rybnik reservoir are of natural origin. These radioisotopes come from radioactive series occurring in nature: thorium and uranium-radium. The primordial radionuclide ^40^ K occurs at the highest level. The radioactivity value of the ^40^ K radionuclide in each sample exceeds 100, sometimes 200 Bq·kg^−1^. Most of it is found in samples taken near the dam and inlet locations: 1, 9 and 4 in amounts of 266.9 ± 6.3, 245.6 ± 9.9 and 206.4 ± 7.4 Bq·kg^−1^, respectively. Approximately slightly lower levels of radioactivity were observed in the samples collected from the central and coastal parts of the reservoir. The level varied between 180 and 190 Bq·kg^−1^ in samples 2, 3, 5 and 6. The lowest radioactivity was found in sample 8.

**Table 1:** Content of radionuclides in bottom sediments

Samples no	Radioactivity A (Bq∙kg^−1^)						
	^137^Cs	^40^ K	^228^Th	^228^Ra	^226^Ra	^210^Pb	^238^U
1	8.58 ± 0.28	266.9 ± 6.3	10.5 ± 1.6	11.3 ± 1.8	11.6 ± 1.8	17.2 ± 3.2	10.0 ± 2.7
2	2.94 ± 0.38	195.7 ± 7.6	6.7 ± 2.4	8.2 ± 2.1	9.22 ± 0.83	25.4 ± 3.0	5.3 ± 2.7
3	1.83 ± 0.31	189.9 ± 6.3	5.0 ± 2.2	4.8 ± 1.7	6.07 ± 0.69	10.8 ± 2.6	6.6 ± 2.3
4	6.45 ± 0.39	206.4 ± 7.4	7.3 ± 2.1	7.2 ± 2.1	9.50 ± 0.83	24.9 ± 3.0	7.1 ± 2.7
5	2.35 ± 0.34	186.3 ± 6.8	6.9 ± 1.2	7.3 ± 1.9	7.04 ± 0.75	17.3 ± 2.8	4.5 ± 2.5
6	1.95 ± 0.24	195.8 ± 6.2	6.8 ± 1.2	7.51 ± 0.92	8.9 ± 1.0	14.7 ± 1.7	6.6 ± 1.4
7	1.55 ± 0.2	152.7 ± 5.8	5.9 ± 1.3	6.3 ± 1.0	7.1 ± 1.3	12.2 ± 1.8	3.2 ± 1.5
8	2.08 ± 0.31	124.8 ± 6.0	7.4 ± 1.1	6.9 ± 1.8	8.79 ± 0.72	19.2 ± 2.6	6.2 ± 2.3
9	44.73 ± 0.68	245.6 ± 9.9	16.6 ± 1.8	16.0 ± 2.9	19.9 ± 1.2	109.9 ± 4.2	17.3 ± 3.6

Similar values of radioactivity were obtained for the radionuclides ^228^Th and ^228^Ra. The highest concentrations were found in samples 9 and 1, respectively, 16.6 ± 1.8 and 10.5 ± 1.6 Bq·kg^−1^ for ^228^Th and 16.0 ± 2.9 and 11.3 ± 1.8 Bq·kg^−1^ for ^228^Ra. For the remaining samples, the values did not exceed 10 Bq·kg^−1^. For every radionuclide from the uranium-radium series, the highest radioactivity was observed in samples 1 and 9 with the highest accumulation in inlet sample 9. Regarding ^226^Ra radionuclide, it was noticed that its level varies significantly in sediment samples. The highest values of the ^226^Ra radioisotope were detected in samples 9 and 1, 19.9 ± 1.2 and 11.6 ± 1.8 Bq·kg^−1^, respectively. For the rest, they were between 7 and 10 Bq·kg^−1^. The same dependence can be observed for ^238^U. The highest ^238^U level was in sample 9 (17.3 ± 3.6 Bq·kg^−1^). For the remaining samples, it was below 10 Bq·kg^−1^ with the lowest level found in sample 7. In the case of the ^210^Pb, the greatest diversity in radionuclide concentration was determined. The highest value was recorded in sample 9, which equals 109.9 ± 4.2 Bq·kg^−1^. In samples 2 and 4, the value of 20 Bq·kg^−1^ was exceeded. For the rest of radioactivity of the samples, the value was less than 20 Bq·kg^−1^.

An artificial radionuclide ^137^Cs was identified in the sediment samples. The concentration of ^137^Cs was in the range of 1.55 ± 0.20–44.73 ± 0.68 Bq·kg^−1^. As in the case of natural radionuclides, the largest accumulation of ^137^Cs was in sample 9. Compared to the sample located on the shore (sample 7), the highest concentration of ^137^Cs is about 20 times higher. The majority of sediment components were sand (over 90%) (Baran et al. 2019) that is why the level of radioactivity of ^137^Cs did not reach a high value. Such composition did not favour ^137^Cs sorption (Ziembik et al. 2010). In Poland, two main sources of ^137^Cs radioisotope are distinguished. These are the Chernobyl accident and nuclear weapons test. The source of the concentration of ^137^Cs in surface samples of sediments comes probably from the Chernobyl accident. It is found in the soils surrounding the reservoir and is also transported with the waters that inflow to the reservoir. The results presented can be compared to other lakes in the Upper Silesian Anthropogenic Lake District. ^137^Cs contamination in Pławniowice Lake is more than twice as high as in Rybnik Lake (Tytła and Kemert 2023). Regarding the natural radionuclide, the radioactivity of ^40^K is also higher. As a fact that soils from the surroundings also constitute a part of sediments, the data from radiological monitoring carried out in Poland can be compared. The level of radioactivity of natural radioisotope in Rybnik reservoir is lower than the average radioactivity in Silesian Voivodship, and also, they are lower than world averages. The measured ^137^Cs deposition in Rybnik is one of the lowest observed in Southern Poland, and the concentrations of this radionuclide in the sediment are lower than the global averages (UNSCEAR 2000; PAA 2023).

### Heavy metal content of sediments and classification

For individual elements, the values also varied, from 67.4 ± 1.2 to 292.8 ± 5.1 mg Zn, from 66.1 ± 3.3 to 138 ± 11 mg Cu, from 6.88 ± 0.33 to 133 ± 12 mg Ni, from 5.28 ± 0.12 to 44.8 ± 1.5 mg Pb, from 0.520 ± 0.030 to 4.020 ± 0.090 mg Cd and from 8.52 ± 0.95 to 37.0 ± 8.1 mg Cr kg^−1^ d.m. (Table 2). The values of heavy metals from the lowest to the highest total amount in the sediments in the studied reservoir are as follows: Cd < Cr < Pb < Ni < Cu < Zn. Taking into account Zn, Cd and Pb in a given sediment, sample 9 was characterised by the highest contents of these heavy metals. The highest content of Ni and Cr was found in sample 7 and Cu in sample 8 (Fig. 2). The results of the sediment samples tested were classified according to geochemical, geoaccumulation and ecotoxicological criteria. Table 3 shows the classification of the bottom sediments of the Rybnik reservoir according to the geochemical criterion used by the Polish Geological Institute. The criteria included elements such as Cd, Cr, Cu, Ni, Pb and Zn in the samples discussed in this paper. The Ni content of sample 7 is the only case where the value of 132.63 mg kg^−1^ is classified as out of grade. This value is about 100 times higher than the remaining nickel values. In other cases, Ni is classified with its values ​in Class I and in Class II with sample number 7. Cu is classified in most cases as Class III and then as Class II. Cr performs best, with each sample classified as Class I, and Pb in most cases has values at background levels. With the exception of one case, all sediment samples meet the criteria for Classes I to III. It can also be noticed that the reservoir is mainly contaminated with Cu. Then, Table 4 presents the classifications based on the geoaccumulation index. Also, Ni in sample 7 was classified as the highest class compared to the other heavy metals tested. It was a Class 5 on a scale from Class 0 to Class 6. Cu also has high values, fitting into Class 4. Class 0 and Class 1 were assigned mainly to the Pb and Cr elements. Considering the sample and not the element, sample no. 9 was characterised by weaker classes. The results of the geochemical indices and the geoaccumulation index were similar to each other and reached similar conclusions. The bottom sediments of the reservoir are largely contaminated with Cu, a deviation was recorded at the same point and low contamination for Pb and Cr was recorded. The evaluation using two indicators is objective and does not show much variation. Table 5 shows the last classification according to ecotoxicological indicators. Ni from sample 7 again has a high content and is the only one classified as level IV (> PEC). The remaining sediments meet the criteria up to level III (> MEC ≤ PEC), which means that the sediments are moderately polluted. However, it should be noted that many elements and samples meet the requirements for level I (TEC). After analysing selected sediment samples, it was also observed that samples around the dam and inlet zones were characterised by the highest toxicity.

**Table 2:** Content of heavy metals in bottom sediments

Metals	Samples no								
	1	2	3	4	5	6	7	8	9
	mg∙kg^−1^ d.m								
Zn	208.5 ± 3.2	128.0 ± 8.2	96.48 ± 2.84	149.5 ± 4.6	67.4 ± 1.2	115.8 ± 4.3	82.7 ± 4.8	109.9 ± 7.5	292.8 ± 5.1
Cu	108.6 ± 1.1	94.8 ± 9.1	74.7 ± 4.0	131.6 ± 2.4	123.8 ± 10.6	113.0 ± 2.1	66.1 ± 3.4	138.2 ± 11.6	18.45 ± 0.83
Ni	11.62 ± 0.11	6.88 ± 0.33	8.15 ± 0.30	8.08 ± 0.21	7.20 ± 0.61	9.87 ± 0.39	132.63 ± 12.50	7.36 ± 0.27	23.47 ± 0.78
Pb	9.41 ± 0.68	7.95 ± 0.33	5.28 ± 0.12	11.36 ± 0.34	8.34 ± 0.20	7.510 ± 0.030	5.92 ± 0.21	7.37 ± 0.29	44.8 ± 1.5
Cd	0.620 ± 0.010	1.260 ± 0.040	0.590 ± 0.010	1.790 ± 0.080	3.68 ± 0.26	0.720 ± 0.011	0.520 ± 0.030	1.62 ± 0.07	4.02 ± 0.09
Cr	12.590 ± 0.020	10.96 ± 0.46	9.47 ± 0.31	10.051 ± 0.050	8.52 ± 0.95	9.780 ± 0.050	37.03 ± 8.14	8.740 ± 0.090	14.87 ± 0.65

**Table 3:**
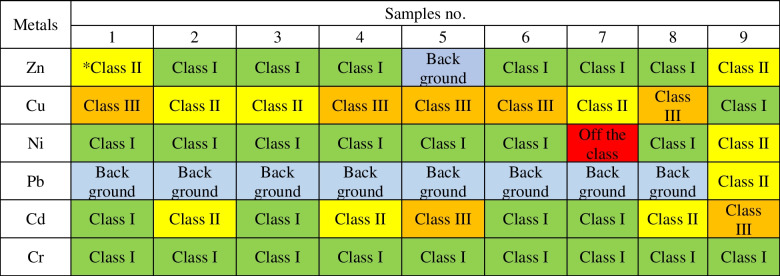
Classification of bottom sediments according to geochemical criteria

*Class I—non contaminated sediments, class II—moderetely uncaontaminated sediments, class III—contaminated sediments

**Table 4:**
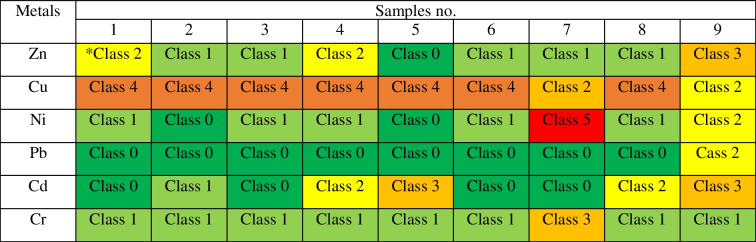
Classification of bottom sediments according to the geoaccumulation index

*Class 0—unpolluted, calss 1—unpolluted to moderately polluted, class 2—moderately polluted, class 3—moderately to strongly polluted, class 4—strongly polluted, class 5—strongly to extremely polluted, class 6—extremely polluted

**Table 5:**
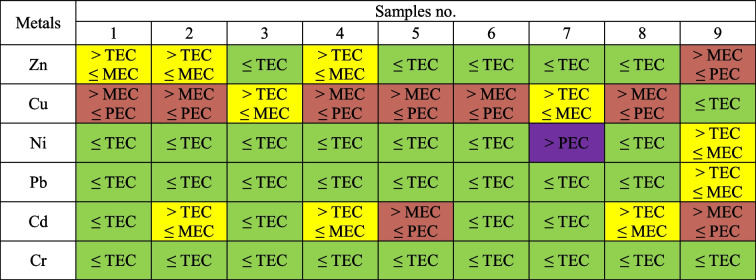
Classification of bottom sediments according to the ecotoxicological criterium

Level I, green; Level II, yellow; Level III, red; Level IV, purple

Most pollutants accumulate in the inlet zones and near the dam. Regarding origin, heavy metals can come from geological changes in a given area, mainly from the occurrence of iron (Szalińska et al. 2010). Natural occurrences may also be related to storm runoff, atmospheric deposition, organic matter, or geological weathering of the Earth’s crust. Their content may also be related to anthropogenic activities such as leaching from landfills, industrial sewage, the use of fertilisers and pesticides on agricultural fields and then washing them into water bodies. Each body of water is located in a different area and is therefore also exposed to different factors. Each water reservoir should be considered individually (Sojka et al. 2019; Muneer et al. 2022). The heavy metals considered may have a common geological origin, but anthropogenically, they come from different sources. For example, according to Sojka and Jaskuła (2022), 45% of Pb came from natural sources and 55% from anthropogenic sources. Of these, 29.2% of pollution is related to urban sources and 39.1% to agricultural areas. There are agricultural lands in the vicinity of the Rybnik reservoir, which are largely in use, and in some of them, ruderal vegetation and birch saplings grow. Ruderal vegetation most often occurs on altered substrates previously used by humans, often remnants of agricultural crops (Miłowski 2020). In the case of Zn, its occurrence is related to wastewater management and products that enter the reservoir after wastewater treatment (Sojka et al. 2019). In the vicinity of the reservoir, in the residential zone, infrastructure is being developed and new residential buildings are being built, which has a significant impact on the increase in the amount of sewage discharged (Miłowski 2020). The occurrence of each tested element is influenced by the presence of hard coal deposits and mining areas on the ground. In some of them, exploitation was suspended, and the areas became a wasteland. Some mining areas show surface deformations, which leads to external damage, including residential areas and further land deformation (Miłowski 2020). General industrial and urban development since 1945 has resulted in an increase in heavy metal content in bottom sediments in water reservoirs and rivers (Dendievel et al. 2022). Some comparisons of reached results can be made. Referring to the data about the Rzeszów artificial reservoir (built in 1973), some relations can be observed (Maj-Zajezierska and Koszelnik 2018). When Zn, Pb and Cd are compared, the grading of heavy metals is the same. The highest content is for Zn, then Pb, and the lowest amount is for Cd. In our studies, Pb ranged from 5.28 to 44.75 mg kg^−1^ d.m. In the Rzeszów reservoir, the Pb content was determined to be from 2.98 to 25.42 mg kg^−1^ d.m. (Maj-Zajezierska and Koszelnik 2018). So, a higher level of this metal occurs in the Rybnik reservoir which may be related to its location in an industrialised place, furthermore exposed to short- and long-range impacts. Considering that the Rybnik reservoir is located in the Silesian Voivodeship, it is worth examining the water reservoirs in the Czech Republic. Water reservoirs do not provide conditions for sediments to move naturally, as occurs in rivers, where water movement is continuous. Hence, the accumulation of bottom sediments in water reservoirs is greater and strongly related to the surroundings. Typical processes related to the structure of the site and unusual processes related to the surroundings occur in reservoirs (Sedláček et al. 2022a). In the Pocheň reservoir in the Czech Republic, the occurrence of Cu, Zn and Pb was analysed. The highest overall concentration of heavy metals was recorded in the central part of the reservoir. The origin of Cu and Zn was considered anthropogenic, from agricultural sources, fertilisers and pesticides due to the agricultural nature of the reservoir. The Pb content in the reservoir is high and constant, related to previous changes (Sedláček et al. 2022a, b). The next reservoirs analysed in the Czech Republic were Hervartov, Nižný Žipov and Byšta. The level of Pb is equal to 26, 24 and 25 mg kg^−1^ d.m. In the presented case, these were values from 5.28 to 44.75 mg kg^−1^ d.m. The Cr content for Czech reservoirs ranged from 85 to 100 mg kg^−1^ d.m. Cu was in the range of 11 to 25 mg kg^−1^ d.m. Zn showed the greatest discrepancy in the range from 56 to 136 mg kg^−1^ d.m. According to the collected data, it was found that the presence of nutrients in sediments does not have an adverse effect on the water quality in reservoirs but there is the risk of remobilisation of pollutants under specific conditions (Junakova et al. 2021). The study by Baran et al. (2019) found a significant correlation between organic matter and the content of metals indicating that organic matter plays an important role in the behaviour of metals in the bottom sediments of the Rybnik reservoir. Organic matter and heavy metals enter the Rybnik reservoir together with municipal wastewater, industrial sewage discharged by the Rybnik power plant and long-range transport associated with the contaminated water of the Ruda River (Baran et al. 2019). It is also worth emphasising that the level of pollution assessment based on the geochemical index seems to be the convenient and standardised approach and is quite often used; however, the use of them can be problematic because the selection of parameters for these indices is arbitrary and thus debatable. For Igeo, the choice of a correct geochemical background is critical, as it will determine the appraisal of the observed contamination (Birch 2017).

## Conclusions

The selected reservoir is situated in a highly urbanised environment and surroundings. It can be concluded that both regional and local scales of impacts in bottom sediments related to anthropogenic activities are rather at an average level. In particular, attention should be paid to the low level of radioactivity in the examined sediments, despite the possible numerous sources of TENORM radionuclide (from coal combustion in the nearest operating power plant or other forms of industry at this urbanised area). This does not mean that there is no impact on surrounding areas. The source of the presence of artificial ^137^Cs is attributed to uncontrolled nuclear accidents or nuclear weapons tests. Its amount is related to the range and distribution after the Chernobyl accident. The decreasing amount of ^137^Cs in the environment is related to migration processes and its radioactive decay. There are no extra sources or processes which increase the level of this radionuclide in the research area.

Assessing the quality of bottom sediments using two different geochemical indicators is objective and does not show large differences in determining their heavy metal contamination. Evaluation of the quality of bottom sediments of the studied reservoir according to ecotoxicological indicators showed that the collected sediments are contaminated to an average degree and meet criterion III (> MEC ≤ PEC). The element that poses a threat to benthic organisms is Ni (spot) and Cu. The content of artificial radionuclides, as well as the geochemical and ecological indicators used, can serve as an indicator of the level of anthropopressure in the vicinity of the Rybnik reservoir. The proposed research scheme and analysis of this facility may be a reference point for other artificial water reservoirs in the world.

## Data Availability

All data analysed during this study are included in this published article. Additional data is available on request.
